# Mapping child health and development policies and implementation challenges in South Africa: A scoping review

**DOI:** 10.4102/jphia.v17i1.1779

**Published:** 2026-06-27

**Authors:** Witness Mapanga, Chenai Mlandu, Khuthala Mabetha

**Affiliations:** 1 DSTI-NRF Centre of Excellence in Human Development, Faculty of Health Sciences, University of the Witwatersrand, Johannesburg, South Africa; 2 SAMRC/Wits Developmental Pathways for Health Research Unit, Department of Paediatrics, Faculty of Health Sciences, University of the Witwatersrand, Johannesburg, South Africa

**Keywords:** child health and development, child support grant, monitoring and evaluation, policy implementation, South Africa

## Abstract

**Background:**

South Africa faces significant health challenges due to a combination of infectious diseases, non-communicable diseases, malnutrition and underdevelopment among children. While policies aimed at improving child health and development have sought to address these issues by focusing on improving healthcare access, nutrition, education and equitable service delivery, gaps in implementation persist.

**Aim:**

To map available literature and policy documents regarding child health and development in South Africa over the last 15 years.

**Setting:**

The review was conducted in South Africa.

**Method:**

The scoping review was conducted using the Joanna Briggs Institute methodology and reported following the Preferred Reporting Items for Systematic Reviews and Meta-Analyses extension for Scoping Reviews. We searched using academic databases (MEDLINE, Google Scholar, African Journals Online [AJOL]), government reports (South African Department of Health, National Planning Commission) and grey literature from 2008 to 2023. All authors were involved in the study selection and data extraction, and over 90% agreement was reached to include relevant articles. Records were screened using predefined inclusion criteria. Data were charted and synthesised thematically to map policy domains and identify implementation patterns and evidence gaps.

**Results:**

Policy attention was highly concentrated in food provision; maternal and child health; child growth and development; economic well-being; and access to resources, though these themes showed uneven articulation and poor-to-moderate implementation. The policy landscape also reflected poor prioritisation and weak articulation in practice for caregiver mental health and child psychosocial well-being. Major barriers include inequitable resource allocation and service delivery across provinces, limited intersectoral collaboration and incomplete monitoring data.

**Conclusion:**

South Africa has developed comprehensive child health and development policies; however, gaps in implementation continue to hinder progress.

**Contribution:**

The findings can help address gaps in the implementation of child health and development policies in South Africa through policy integration, equitable resource distribution and improved data monitoring to meet child health and development goals.

## Introduction

Over the past 15 years, South Africa has implemented a wide range of policies and programmes aimed at improving child health and development.^1^ These efforts are embedded within the broader context of the country’s socioeconomic challenges, health disparities and the urgent need to improve the overall well-being of children. Despite economic growth and advances in healthcare, South Africa still faces the dual burden of addressing infectious diseases such as human immunodeficiency virus (HIV) and/or acquired immunodeficiency syndrome (AIDS) while also managing non-communicable diseases, malnutrition and underdevelopment among children.^2^ Policies targeting child health and development have sought to address these issues by focusing on nutrition, education, healthcare access and equitable service delivery.^3,4^ However, significant gaps remain, especially in policy implementation, monitoring and evaluation.

Child health in South Africa is shaped by structural determinants such as poverty, geographic inequities and limited healthcare infrastructure – particularly in rural and underserved communities. In response, several major policy frameworks have been introduced over the past decade to strengthen service delivery and promote child well-being.^5^ Adopted in 2012 by the South African government, the National Development Plan (NDP) 2030 outlines the country’s long-term development vision, emphasising universal health coverage and reducing child mortality.^1^ Complementing this, the Strategic Plan for Maternal, Newborn, Child and Women’s Health (MNCWH) and Nutrition prioritises a continuum of care from pregnancy through early childhood.^3^

Additional policies, including the Integrated School Health Policy (ISHP),^6^ the National Health Insurance (NHI) initiative,^7^ the Expanded Programme on Immunisation (EPI),^6,8^ Early Childhood Development (ECD) Policy^4^ and the National School Nutrition Programme (NSNP)^9^ highlight the government’s commitment to improving children’s developmental trajectories.

According to the World Health Organization (WHO) informed conceptual framework, the effectiveness of a policy relies not only on its design but also on the institutional systems that enable its implementation.^10^ Although numerous child health and development policies have been introduced in South Africa, it remains unclear whether these expanding policy commitments are being matched by improvements in governance and service delivery. Persistent structural inequalities, budget limitations and uneven provincial capacity continue to shape policy implementation, often creating a gap between national priorities and the realities of service delivery on the ground.^11^

Although South Africa has comprehensive and progressive child health and development policies, available funding continues to prioritise specific programmes and individual health outcomes rather than supporting a broader systems-level approach that examines how national policy intentions align with decentralised implementation structures. Consequently, the reasons behind persistent service-delivery gaps remain difficult to pinpoint. These gaps may stem from weaknesses in policy design, limitations within provincial governance and administrative capacity or wider structural pressures affecting the health sector.^12,13^

This scoping review addresses this gap by examining the evolution of child health and development policies alongside the governance and implementation contexts that shape their delivery. By adopting a systems-oriented lens, the review moves beyond descriptive mapping to analyse the relationship between policy ambition and implementation capacity in South Africa, highlighting gaps that need addressing to meet national and international goals (such as the sustainable development goals).^14^

## Methods

### Study design

This study employed a scoping review methodology to systematically map child health and development policies and related implementation evidence in South Africa. The review was conducted according to the Joanna Briggs Institute guidelines and is reported following the Preferred Reporting Items for Systematic Reviews and Meta-Analyses (PRISMA) extension for Scoping Reviews (ScR).^15^ The purpose was to map available literature and policy documents regarding child health and development in South Africa over the last 15 years. The review consisted of five stages: (1) identifying the research question, (2) identifying relevant studies and policies, (3) study and policy selection, (4) charting the data and (5) collating, summarising and reporting the results.

### Data sources and search strategy

**FIGURE 1:**
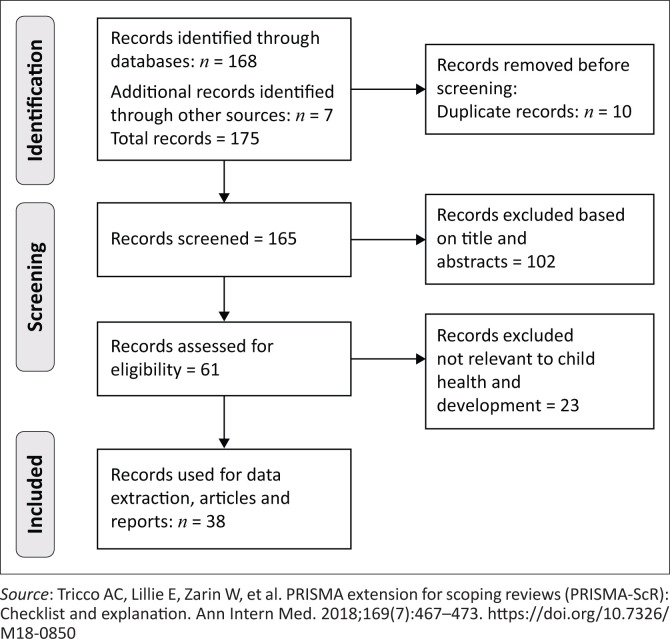
Preferred Reporting Items for Systematic Reviews and Meta-Analyses extension for Scoping Reviews flow diagram for South Africa child and health policy.

A comprehensive literature search was conducted in the following sources: (1) academic databases: MEDLINE, Google Scholar, African Journals Online (AJOL), (2) government reports: South African Department of Health, National Planning Commission, and (3) grey literature: reports from the United Nations Children’s Fund (UNICEF), WHO and non-governmental organisations. A combination of keywords and Boolean operators was used to identify relevant literature. Key terms included: (‘child health’ OR ‘childhood development’) AND (‘policy’ OR ‘strategy’ OR ‘framework’ OR ‘implementation’) AND ‘South Africa’. Filters applied: date range (2008–2023), study design (policy reviews, empirical studies) and language (English). The date range was selected to include studies that reflect current policies and contemporary evidence following the introduction of key policies, ensuring relevance to current practice and maintaining feasibility and rigour. Three reviewers conducted the screening and selection, resolving disagreements through consensus or consulting an expert reviewer. The study selection process is presented in a PRISMA-ScR flow diagram (Figure 1).^15^ The flow diagram clearly represents the volume of evidence identified and the screening stages applied, enhancing the transparency and rigour of the review process.

For independent review, all the authors were involved in the screening process and conducted study selection and data extraction by examining study titles and abstracts to identify studies that potentially met the inclusion criteria. The authors conducted a rigorous selection to ensure that the studies selected aligned with the scoping review’s research question and scope. A structured process was undertaken to reach an agreement, and over 90% of the agreement included relevant articles.

### Population

Children and caregivers, including maternal and household contexts relevant to child health and development in South Africa.

### Eligibility criteria

#### Inclusion criteria

The following were the inclusion criteria applied: (1) peer-reviewed articles, government policy documents, strategic plans, reports and grey literature on child health and development between 2008 and 2023; and (2) English-language sources.

#### Exclusion criteria

The following were the exclusion criteria applied: (1) policies outside of South Africa, (2) non-English sources, and (3) not relevant to child health and development.

### Data extraction

A standardised data extraction form was used to capture policy details (e.g. name, year, scope and impact), key health indicators and findings on child health and development. The data extraction form was developed based on the key indicators (Appendix 1) that were targeted for review in this scoping review.

### Data synthesis

Data were synthesised qualitatively and quantitatively – a thematic synthesis approach was employed to analyse and interpret the included sources. Initial codes were developed inductively from both policy documents and empirical studies, focusing on key policy domains and features related to implementation. Through an iterative process of comparison and collaborative discussion, these codes were consolidated into broader thematic categories. Policy documents were mainly analysed to understand policy intent and design features, while peer-reviewed empirical studies provided contextual insights into how policies were interpreted and implemented in practice, including challenges reported in different settings. Instead of hierarchically weighting sources, the synthesis drew on the complementary contributions of each to build a comprehensive, system-level picture of child health and development policy implementation in South Africa.

Policy areas were categorised into areas, such as food provision; maternal, newborn and child health, child growth and development; economic well-being; caregiver mental health and well-being; child psychosocial well-being; and access to resources. Each policy area was graded based on the (1) availability of prevalence data and (2) policy implementation (see Table 1).^16^

**TABLE 1:** Criteria used to grade policy area.

Grade	Prevalence data	Policy and implementation
A	Published national and regional data available disaggregated for this age group	National implementation for more than 10 years with support structures
B	Published national and regional data, but not specific to this age group	National implementation with less than 5 years, with minimal support structures
C	Only regional prevalence data for this age group	National policies have been proposed but not implemented or have minimal support structures
D	Only regional prevalence data, but not specific to this age group	No national policies
E	No prevalence data	-

*Source:* Draper CE, Tomaz SA, Harbron J, Kruger HS, Micklesfield LK, Monyeki A, et al. Results from the Healthy Active Kids South Africa 2018 Report Card. S Afr J Child Health. 2019;13(3):130–136.

## Review findings

In summary, the matrix in Table 2 shows that while most domains are policy-rich, implementation remains poor and uneven across provinces.^13^ Caregiver mental health and child psychosocial well-being are particularly underdeveloped, with limited policy articulation and weak execution. In contrast, economic well-being is the only domain showing consistently strong implementation due to more established administrative systems. Access to essential resources remains constrained by persistent structural inequalities, undermining the effectiveness of otherwise well-designed national policies.

**TABLE 2:** Policy implementation matrix.

Policy area	National policy intent	Provincial implementation evidence	Observed impact	Monitoring and evaluation capacity	Classification	Grades (data/ implementation)
Food provision	NSNP provides daily school meals to improve nutrition, attendance and learning	Delivery is widespread, but quality and infrastructure vary; rural schools are disadvantaged	Improved attendance; uneven nutrition outcomes	Moderate – GHS food security data, but missing age-specific indicators	Policy-rich, uneven implementation	B/B
Maternal, newborn and child health	Continuum of care and routine immunisation	Provincial disparities; IMCI not standard; weak referral systems	Decline in mortality where coverage is strong; uneven progress	Strong national and/or regional indicators; weaker district reporting	Policy-rich, implementation-strained	A/B
Child growth and development	Growth monitoring and early detection of undernutrition	Limited school-based monitoring; chronic diseases are under-addressed	Stunting persists; detection is limited by capacity	National and/or regional data strong; local tracking uneven	Policy-rich, low implementation	A/B
Economic or material well-being	CSG reduces child poverty and supports households	National reach high; consistent implementation	Poverty reduction, but no link to long-term empowerment	Robust administrative data	Policy-rich, moderate implementation	A/B
Caregiver mental health	Included in national mental health framework; no standalone caregiver policy	Minimal provincial rollout; rural gaps prominent	High caregiver stress; limited access to services	Sparse indicators; weak routine tracking	Policy-poor, implementation-poor	C/C
Child psychosocial well-being	Fragmented intent; partial support	Uneven, poorly coordinated services	Persistent exposure to violence and stress; limited interventions	Very limited routine indicators	Policy-poor, implementation-poor	C/C
Access to resources	Strong commitments to housing, water, sanitation, electricity	Large provincial and rural–urban disparities	Incremental improvements: major deficits persist	Strong survey data; inconsistent local service data	Policy-rich, implementation-poor	A/C

*Source:* Department of Planning, Monitoring and Evaluation. National Evaluation Policy Framework (NEPF). Pretoria (ZA): Government of South Africa; 2019.

CSG, child support grant; GHS, general household survey; IMCI, integrated management of childhood illness; NSNP, National School Nutrition Programme.

### Food provision

The NSNP plays a crucial role in improving child nutrition, school attendance and cognitive performance among over nine million learners in South Africa. The programme ensures that children receive at least one meal per school day, which helps alleviate short-term hunger and supports overall health. However, inconsistencies in meal quality and infrastructure challenges in food storage and preparation limit the programme’s effectiveness. Additionally, food security remains a significant concern, especially outside the school environment, where many children still experience inadequate access to nutritious food (Table 2). Using the current evidence, a grade B has been assigned to food provision for the availability of prevalence data.^5^ Although national, available and regional data from the latest general household survey (GHS) on food security are available, they do not cover all age groups of children, particularly those aged 5 years and above. A grade B has been assigned for policy and implementation, as the policy faces implementation inconsistencies in urban-rural settings^17,18^ (Table 2).

### Maternal, newborn and child health

The Strategic Plan for MNCWH and Nutrition outlined priority interventions to ensure every mother and child in South Africa receives a comprehensive package of services to reduce maternal, newborn and child mortality and illness. These interventions include services such as antenatal care, improved access to care during labour, intrapartum care, postnatal care of the mother and newborn within 6 days of delivery, and immunisation. However, the successful implementation of these interventions continues to be hampered by substandard care, such as the uneven distribution of appropriately skilled healthcare workers, obstetric care for urban versus rural areas and the public versus private health system.^3^ Given the current evidence, a grade A has been assigned for the availability of prevalence data because the most recent data for maternal, newborn and child health indicators are available at the national and regional levels.^19^ However, a grade B has been assigned for policy and implementation because the policies (MNCWH, Nutrition and EPI) face implementation challenges across provinces and districts, particularly in rural settings^3,13^ (Table 2).

### Child growth and development

Efforts to reduce child malnutrition and end under-five year old mortality have led to policies focused on growth monitoring to assess child growth and development. Key indicators such as height, weight and mid-upper arm circumference (MUAC) highlight ongoing concerns regarding stunting and undernutrition. Despite national initiatives, chronic conditions such as childhood diabetes and obesity receive insufficient policy attention. Furthermore, there is a lack of comprehensive school-based growth-monitoring systems, limiting early detection and intervention for undernourished children (Table 2). Based on the current evidence, a grade A has been assigned for the availability of prevalence data because national and regional data are available for the indicators of child growth and development.^5^ However, a grade B has been assigned for policy and implementation, as the policies face implementation challenges in under-resourced settings^11^ (Table 2).

### Economic and material well-being

Financial relief measures, particularly the Child Support Grant (CSG), serve as essential poverty alleviation tools, supporting children in low-income households. The grant ensures that children have access to necessities, reducing financial stress on families. However, long-term economic stability remains unaddressed because income support policies are not integrated with broader child development strategies. Additionally, there are no substantial mechanisms to transition grant recipients towards sustainable financial independence, leaving families reliant on continued aid (Table 2). Using the current evidence, a grade A has been assigned for the availability of prevalence data because the most recent data at the national and regional levels are available for economic and material well-being.^5^ Although the CSG policy is implemented across all regions, a grade B has been allocated for policy and implementation because there is a lack of integration of income support policies with broader child development strategies and long-term economic stability policies for families^20^ (Table 2).

### Caregiver mental health and well-being

Caregivers play a critical role in child health and development, yet many experience high levels of stress and depression, especially in disadvantaged households. While some policies provide access to mental health services, such access remains limited, particularly in rural areas. The lack of a structured support system for caregivers impacts both their well-being and their ability to provide adequate care for children. Strengthening caregiver-focused mental health policies and ensuring widespread availability of support structures are necessary to improve overall family stability (Table 2). Using current evidence, a grade C has been assigned for the availability of prevalence data, as there is a lack of data on caregiver mental health indicators.^21^ A grade C has been assigned for policy and implementation because the current proposed policy is not a standalone policy solely dedicated to caregivers^22^ (Table 2).

### Child psychosocial well-being

Exposure to economic hardship, violence and instability negatively affects children’s mental and emotional well-being. Some policies address psychosocial support and resilience-building; however, they remain fragmented and inadequately integrated into broader child health programmes. There is insufficient attention to the long-term psychological effects of poverty and violence, leaving many vulnerable children without proper mental health interventions. Strengthening policies that promote resilience and mental health support is essential to mitigate these adverse effects (Table 2). Based on current evidence, a grade C has been assigned for the availability of prevalence data because regional data on child psychosocial well-being indicators are lacking.^16^ A grade C has been allocated for policy and implementation because the policies have minimal support structures and poor implementation^10,16^ (Table 2).

### Access to resources

Basic infrastructure, including safe housing, clean water, electricity and sanitation, remains a crucial determinant of child well-being. Although housing policies have improved access in some areas, significant disparities persist, particularly in rural and low-income communities. Limited policy focuses on resource insecurity, which further exacerbates challenges in child development, as inadequate living conditions contribute to poor health outcomes. Ensuring equitable access to essential services is necessary for closing infrastructure gaps and improving overall child well-being (Table 2). Using current evidence, a grade A has been assigned for the availability of prevalence data because national and regional data for access to resources indicators are available from national surveys.^5,23^ However, a grade C has been allocated for policy and implementation because access to basic services like housing, water, electricity and sanitation remains a challenge in rural South Africa, with significant disparities compared to urban areas^1,5^ (Table 2).

## Discussion

The review highlights key policy developments, achievements and implementation challenges in child health and development in South Africa. This scoping review shows that South Africa’s policy environment for child health and development is extensive and conceptually aligned with global child well-being frameworks. Yet across all seven domains, implementation quality remains uneven, reflecting deeper structural challenges. Rather than a lack of policy intent, the findings reveal systemic constraints stemming from governance arrangements, financing flows, accountability mechanisms and sub-national capacity. These structural drivers shape how effectively policies translate into equitable service delivery.

A major factor shaping these implementation patterns is South Africa’s decentralised governance structure, in which national departments develop policies while provincial governments are responsible for implementing them. Although this arrangement allows provinces to tailor implementation to local realities, it also heightens disparities when provincial capacity differs. Provinces vary widely in administrative strength, budget management, workforce availability and managerial oversight. District health systems – intended to coordinate service delivery – often operate with limited financial and technical support, constraining their ability to translate national priorities into routine practice. Municipalities, which oversee essential services such as water, sanitation and housing, continue to face chronic infrastructure deficits and revenue limitations, further slowing progress in the access-to-resources domain.^12,13^

These structural dynamics help clarify why several policy domains present as policy-rich, yet remain weak in implementation. For example, although the food provision, MNCWH and child-growth domains are supported by well-established national strategies, their execution is constrained by fragmented financing arrangements, dependence on provincial supply chains and uneven capacity at facility level.^3^ Psychosocial and caregiver mental health services face even more limited progress, not only due to the absence of comprehensive policy frameworks, but also because effective implementation requires sustained coordination across the health, education and social development sectors – an area where cross-sectoral collaboration remains insufficient.

Financing arrangements present an additional structural barrier. Many child-and health-focused policies are not supported by conditional grants, forcing provinces to weigh them against competing priorities such as acute care, HIV and tuberculosis (TB) programmes and emergency services. As a result, prevention-oriented and community-based interventions – particularly psychosocial support, school health services and growth monitoring – tend to be chronically underfunded. In the absence of stable and predictable financing, these programmes remain vulnerable to shifts in political leadership, fiscal constraints and changing provincial agendas.^24^

Accountability mechanisms are similarly weak. Although national departments set policy expectations, they often lack clear levers to enforce compliance or ensure minimum service standards across provinces. Monitoring and evaluation systems are fragmented, with some domains (e.g. immunisation, poverty indicators) benefiting from strong national surveys, while others (mental health, psychosocial well-being, service quality) rely on sporadic studies and non-routine reporting. This reinforces an implementation architecture, in which policy visibility is high, but actionable feedback loops are weak, limiting the system’s ability to identify bottlenecks and course-correct.^11,25^

Political economy dynamics also play a significant role in shaping implementation outcomes. Child-focused programmes often compete for limited public funding with more politically visible infrastructure projects and curative health services, which tend to attract greater attention from decision-makers. Although the importance of intersectoral collaboration is widely recognised, translating it into practice is challenging in contexts where government departments operate with separate budgets, performance targets and institutional priorities. These constraints are further compounded by historical inequalities, deeply embedded bureaucratic practices and local power dynamics, all of which influence which programmes receive consistent support and resources.^12^

Despite these challenges, South Africa’s experience offers important lessons for other low- and middle-income countries operating within decentralised governance systems. Many countries face similar tensions between well-defined national policy frameworks and inconsistent delivery at sub-national levels.^26^ The findings of this review emphasise that effective child health and development policies depend not only on strong technical design, but also on the broader governance, financing and accountability arrangements that support implementation. Where systems lack mechanisms for joint planning, aligned budgeting and district-level authority, multisectoral child development agendas are difficult to operationalise in practice. South Africa’s experience highlights the importance of strengthening provincial and district capacity, clarifying institutional mandates and investing in routine data systems to enable more consistent and coordinated implementation across levels of government.

The review suggests that improving child health and development outcomes in South Africa will require reconfiguring elements of the policy implementation architecture rather than making isolated programme-level adjustments. Key opportunities for reform include: (1) establishing clearer vertical accountability structures between national and provincial levels, underpinned by enforceable minimum service standards; (2) developing cross-sector financing mechanisms or pooled budgets that better support integrated child development strategies; (3) increasing the authority and resourcing of district health systems and local governments to improve frontline delivery; and (4) institutionalising multisectoral planning platforms with shared performance indicators and joint targets. Without these system-level reforms, even well-designed national policies are likely to continue encountering implementation bottlenecks.

Overall, the findings indicate that South Africa’s core challenge is not insufficient policy development, but the misalignment between policy ambition, governance structures and implementation capacity. Addressing this gap requires realistic, prioritised reforms focused on strengthening provincial delivery systems, establishing effective intersectoral coordination mechanisms, improving financing predictability and investing in integrated, routine monitoring systems. These foundational changes are essential for translating South Africa’s strong policy commitments into sustainable and equitable improvements in child health and development.

### Implications and recommendations

Given these challenges, several key policy recommendations can be made. The review highlights the need for targeted and achievable reforms to strengthen policy implementation for child health and development in South Africa. Priority actions include enhancement of provincial delivery capacity by establishing minimum service standards, providing targeted technical support, and clarifying accountability mechanisms between national and provincial levels. Improved intersectoral collaboration is also essential, particularly through formalised joint planning platforms that bring together health, education, social development and local government sectors. Strengthening monitoring and evaluation systems – such as integrated child data platforms and routine tracking of caregiver mental health and psychosocial indicators – would further support more responsive implementation. To advance child health outcomes, policies should reinforce school-based nutrition and growth-monitoring services, expand community-level psychosocial and caregiver mental health interventions, link the CSG to livelihood and skills-building initiatives and prioritise infrastructure investment in rural and underserved areas. Achieving these reforms requires coordinated efforts across government sectors, adopting a child-centred approach to service integration, investing in reliable data for monitoring and accountability and aligning budgets with evidence-based priorities while tailoring global guidance to local realities. Collectively, these system-level shifts – supported by improved financing alignment, stronger district authority and more coordinated service delivery – offer practical pathways for translating South Africa’s strong policy ambitions into equitable and effective implementation.

### Limitations of the study

This scoping review has several limitations. Firstly, the analysis draws on publicly accessible policy documents and grey literature, which may introduce bias, as these sources often present programmes favourably and may lack methodological detail. Secondly, routine data systems remain weak in key domains, including caregiver mental health, psychosocial well-being and service quality, restricting the ability to assess these areas comprehensively. Finally, because the review synthesised a diverse body of literature rather than evaluating programme effectiveness directly, the findings describe patterns of policy design and implementation rather than causal impacts.

## Conclusion

South Africa has developed a comprehensive set of policies aimed at enhancing child health and development, yet persistent gaps between policy design and implementation capacity continue to hinder progress. Structural barriers – including decentralised governance arrangements, uneven provincial capacity, fragmented financing mechanisms and weak accountability systems – shape how national policy intentions are translated into services for children and caregivers. Addressing these systemic constraints will require strengthening provincial delivery systems, improving coordination across levels of government, investing in routine and integrated data systems, and advancing caregiver and psychosocial support services that remain insufficiently developed. By prioritising these system-level reforms, South Africa can move closer to aligning its strong policy commitments with effective implementation and achieving more equitable, sustainable improvements in child health and development.

## Appendix 1

**TABLE 1-A1:** Indicators to monitor child and caregiver health status.

Domain	Indicators	Questions on the questionnaire
1. Food provision	1.1. Number of meals per day 1.2. Food security 1.3. Healthy food eaten Fruit & vegetable intake Protein intake 1.4. NSNP – National School Nutrition Programme	1.1. Does your child eat three meals a day? **[D2.5]** 1.2. Is there enough food for your child to eat at every meal? **[D2.4]** 1.3. Healthy food eaten Does your child eat a protein (fish, chicken, meat, eggs, peanut butter) at least 2 × a week? **[D2.2]** Does your child eat vegetables at least 2 × a week? **[D2.3]** 1.4. Does the child eat a meal provided by the primary school nutrition scheme? **[SECTIONDQ7]**
2. Maternal, newborn and child health	2.1. Antenatal care 2.2. Delivery by a skilled attendant 2.3. Postnatal care within 6 weeks 2.4. Low birth weight (< 2.5 kg) 2.5. Children fully immunised under 1 year	2.1. Did you see anyone for antenatal care for this pregnancy? **[412]** 2.2. Who assisted with the delivery of (NAME IN 407)? **[434]** 2.3. How long after delivery did the first check take place? **[465]** How long after delivery was (NAME IN 407)’s health first checked? **[453]** 2.4. How much did (NAME IN 407) weigh? **[442]** 2.5. In addition to what is recorded on (this document/these documents), did (NAME IN 503) receive any other vaccinations, including vaccinations received in campaigns or immunisation days or child health days? **[512]**
3. Child growth and development	3.1. Health status Height Weight MUAC History of medical problems Overweight/underweight Stunting & undernutrition 3.2. Participation in extra-curricular activities	3.1. Health status Child’s height **[SECTIONEQ2]** Child’s weight **[SECTIONEQ1]** Is the child’s mid-upper circumference (MUAC) below 11.5 cm? **[SECTIONEQ3]** Is the child on HIV treatment? **[SECTIONEQ7]** Is the child on TB treatment? **[SECTIONEQ8]** Does the child have diabetes? **[SECTIONEQ9]** Does the child have a history of cardiac conditions? **[SECTIONEQ10]** Does the child have any respiratory conditions (pneumonia, asthma)? **[SECTIONEQ12]** Did/does the child have a confirmed/diagnosed mental health condition? **[SECTIONEQ14]** Did/does the child have seizures? **[SECTIONEQ13]** 3.2. Does the child participate in sporting, cultural, spiritual/religious, arts or recreational activities outside school hours? **[D3.6]**
4. Economic/material well-being	4.1. Access to a grant 4.2. Access to income 4.3. Employment status	4.1. Does this child receive a CSG? **[SECTIONAQ15a]** 4.2. Access to income In addition to the grant, does the family have access to other sources of income? **[D4.1]** Are you able to save a portion of your income/money? (e.g. are you part of a savings club) (like a stokvel)? **[D4.3]** 4.3. Employment status Employment status of parent/caregiver **[SECTIONAQ17]**
5. Caregiver mental health & well-being	5.1. Presence of depression symptoms 5.2. Exposure to stressors 5.3. Access to support structures 5.4. Access to social services 5.5. Access to family support network structures 5.6. Access to food relief	5.1. Depression scale categorised into severe, moderate and no depressive symptoms **[SECTIONCQ1-10]** 5.2. Exposure to stressors: Employment status of parent/caregiver (Unemployment) **[SECTIONAQ17]** In addition to the grant, does the family have access to other sources of income? **[D4.1]** Are you able to save a portion of your income/money? (e.g. are you part of a savings club) (like a stokvel)? **[D4.3]** Do you struggle with paying off debts? (indebtedness) **[D4.5]** Does your child ever go to sleep hungry? **[D2.1]** (children are going hungry). Has the child seen people that are fighting, swearing or hurting each other at home, school or in the community? **[D5.5]** (children’s exposure to violence in home and community); 5.3. Access to support structures Is there anyone in your household/family or community to support you in times of need? **[SECTIONAQ19]** (lack of support); Are there relatives in the household who help with care of the child/rent? **[SECTIONAQ18]** (lack of support with childcare and support in home and community). Is there someone in your home who helps your child with homework? **[D1.4]** (- lack of support network) 5.4. Access to social services After answering questions during the study at the end of last year, were you referred by a social worker to another organisation for support? **[SECTIONCQ5]** 5.5. Access to family support network structures Who is the child living with NOW? **[SECTIONAQ15]** 5.6. Access to food relief What type of service were you referred to? **[SECTIONCQ5a]** Food relief/parcel Mental health support Other services (e.g. vaccinations, eye tests, hearing, education psychologist) Did you receive the service? (e.g. did you get the food; did you get a vaccination, etc.?) **[SECTIONCQ5b]**
6. Child psychosocial well-being	6.1. Strengths & difficulties 6.2. Resilience 6.3. Exposure to stress 6.4. Access to resources	6.1. Strengths and difficulties Scale Strength and Difficulties Questionnaire (SDQ) **[SECTIONFQ2]** Does the child seem sad or depressed? **[SECTIONDQ15]** 6.2. Resilience Scale Child and Youth Resilience Measure (CYRM) **[SECTIONFQ1]** 6.3. **Exposure to stressors** Personal life stressors: Financial difficulties. In addition to the grant, does the family have access to other sources of income? **[D4.1].** Are you able to save a portion of your income/money? (e.g. are you part of a savings club) (like a stokvel)? **[D4.3].** Do you struggle with paying off debts? (indebtedness) **[D4.5]** **Health-related stressor** Child’s height **[SECTIONEQ2]** Child’s weight **[SECTIONEQ1]** Is the child’s mid-upper circumference (MUAC) below 11.5 cm? **[SECTIONEQ3]** Is the child on HIV treatment? **[SECTIONEQ7]** Is the child on TB treatment? **[SECTIONEQ8]** Does the child have diabetes? **[SECTIONEQ9]** Does the child have a history of cardiac conditions? **[SECTIONEQ10]** Does the child have any respiratory conditions (pneumonia, asthma)? **[SECTIONEQ12]** Did/does the child have a confirmed/diagnosed mental health condition? **[SECTIONEQ14]** Did/does the child have seizures? **[SECTIONEQ13]** **Protection and care stressors** Has the child seen people that are fighting, swearing or hurting each other at home, school or in the community? **[D5.5]** Does the child fight with other children? **[SECTIOND21]** Has the child been a victim of abuse or violence at home, in the community or at school? **[D5.6]** Is there evidence of child abuse and/or neglect? **[SECTIONDQ23]** Is there evidence of abuse? **[SECTIONDEQ16]** Is the child well cared for and looks neat and clean? **[SECTIONDQ11]** Compared to other children of their age, does the child have difficulty learning or remembering things or concentrating on an activity they enjoy? **[SECTIONDQ4]** Exposure to violence and abuse; hunger; care arrangements of the child; learning difficulties; child fight with other children Does your child eat three meals a day? **[D2.5]** Is there enough food for your child to eat at every meal? **[D2.4]** Access to social support (caregiver) Are there relatives in the household who help with care of the child/rent **[SECTIONaQ18]** Is your child progressing with their schoolwork? **[D1.2]** Is there someone in your home who helps your child with homework? **[D1.4]** 6.4. **Access to resources** Does your child have a mattress or bed in the house where he/she sleeps every night? **[D4.4]** Do you live in a home that protects you from wind and rain? **[D4.5]** Do you live in a home that has access to clean drinking water? **[D4.6]** Do you live in a home with electricity? **[D4.7]** Do you have a toilet with running water on your property/do you have access to a toilet with running water in your home/property/ yard? **[D4.8]**

*Source:* Patel L, Pillay J, Henning E, Telukdarie A, Norris S, Graham L, et al. Community of Practice for Social Systems Strengthening to Improve Child Well-Being Outcomes. Pretoria, South Africa: UNICEF South Africa; 2021; Department of Health, Statistics South Africa, Medical Research Council & ICF. South African demographic health survey 2016. Key Indicator Report. Pretoria: DOH, Stats SA, MRC & ICF; 2017.
